# Induction of labor compared to expectant management in term nulliparas with a latent phase of labor of more than 8 hours: a randomized trial

**DOI:** 10.1186/s12884-019-2602-2

**Published:** 2019-12-11

**Authors:** Patrick Naveen Sargunam, Lindy Li Mei Bak, Peng Chiong Tan, Narayanan Vallikkannu, Mat Adenan Noor Azmi, Syeda Nureena Zaidi, Sandar Tin Win, Siti Zawiah Omar

**Affiliations:** Department of Obstetrics and Gynaecology, Faculty of Medicine, University of Malaya, Lembah Pantai, 50603 Kuala Lumpur, Malaysia

**Keywords:** Cesarean, Vaginal delivery, Latent phase of labor, Induction of labor, Expectant management, Term, Nullipara, Patient satisfaction

## Abstract

**Background:**

Prolonged latent phase of labor is associated with adverse maternal and neonatal outcomes. Preliminary data indicate that labor induction for prolonged latent phase may reduce cesarean delivery. We performed a study powered to Cesarean delivery to evaluate labor induction compared to expectant management in full term nulliparas hospitalized for persistent contractions but non-progressive to established labor after an overnight stay.

**Methods:**

From 2015 and 2017**,** nulliparas, ≥ 39 weeks’ gestation with prolonged latent phase of labor (persistent contractions after overnight hospitalization > 8 h), cervical dilation ≤3 cm, intact membranes and reassuring cardiotocogram were recruited. Participants were randomized to immediate induction of labor (with vaginal dinoprostone or amniotomy or oxytocin as appropriate) or expectant management (await labor for at least 24 h unless indicated intervention as directed by care provider). Primary outcome measure was Cesarean delivery.

**Results:**

Three hundred eighteen women were randomized (159 to each arm). Data from 308 participants were analyzed. Cesarean delivery rate was 24.2% (36/149) vs. 23.3%, (37/159) RR 1.0 95% CI 0.7–1.6; *P* = 0.96 in induction of labor vs. expectant arms. Interval from intervention to delivery was 17.1 ± 9.9 vs. 40.1 ± 19.8 h; *P* < 0.001, intervention to active labor 9.6 ± 10.2 vs. 29.6 ± 18.5 h; *P* < 0.001, active labor to delivery 7.6 ± 3.6 vs. 10.5 ± 7.2 h; *P* < 0.001, intervention to hospital discharge 2.4 ± 1.2 vs. 2.9 ± 1.4 days; *P* < 0.001 and dinoprostone use was 19.5% (29/149) vs. 8.2% (13/159) RR 2.4 95% CI 1.3–4.4; *P* = 0.01 in IOL compared with expectant arms respectively. Intrapartum oxytocin use, epidural analgesia and uterine hyperstimulation syndrome, postpartum hemorrhage, patient satisfaction on allocated intervention, during labor and delivery and baby outcome were not significantly different across trial arms.

**Conclusions:**

Induction of labor did not reduce Cesarean delivery rates but intervention to delivery and to hospital discharge durations are shorter. Patient satisfaction scores were similar. Induction of labor for prolonged latent phase of labor can be performed without apparent detriment to expedite delivery.

**Trial registration:**

Registered in Malaysia National Medical Research Register (NMRR-15-16-23,886) on 6 January 2015 and the International Standard Randomised Controlled Trials Number registry, registration number ISRCTN14099170 on 5 Nov 2015.

## Introduction

The conventional understanding is that latent phase of labor can last for 20 h and begins when the cervix starts to dilate from 0 to 3 cm or 4 cm and contractions get stronger [1]. More recently according to National Institute of Clinical Excellence UK, the latent phase starts when there are contraction pain, not necessarily continuous accompanied by some cervical effacement and dilation up to 4 cm [2]. Women experienced their onset of labour in a variety of ways and a large proportion of these experiences bore no resemblance to the classical diagnosis of labor and most were unrelated to the duration of labor [3].. Recent studies showed that in predominantly white, healthy nulliparas, duration of the latent phase of labor was a median duration 9.0 h and mean 11.8 h [4] and self-reported prolonged latent phase that lasted 18 h or more occurred in 29.2% of nulliparas [5]. In Chinese nulliparas, singleton term gestation, spontaneous onset of labor, vaginal delivery, and a normal perinatal outcome, latent phase of labor was 5.1 ± 3.2 h [6]. There was no consensus on the definition of a prolonged latent phase of labor or even of labor onset in the research literature [7].

Friedman [8] introduced the relationship between duration of labor and cervical dilatation as a sigmoid curve [1]. More recently, Zhang et al. noted that the pattern of labor progression among nulliparous women in contemporary practice differed significantly from Friedman’s curve with the transition from latent to active phases of labor appearing more gradually [9]. Recently, it has been recognised that the active phase often did not start until at least 6 cm [10] but women whose dilation time from 4 cm to 6 cm exceeds the 90th percentile have increased odds of cesarean delivery and postpartum complications [11]; in nulliparous labor induction cesarean delivery (for failed induction) should not be undertaken during the latent phase prior to at least 15 h after oxytocin and rupture of membranes have occurred [12].

Prolonged latent phase was independently associated with an increased incidence of subsequent labor abnormalities, need for cesarean delivery, depressed Apgar scores, and need for newborn resuscitation [13], and also with oxytocin augmentation, thick meconium staining and admission to the neonatal unit [14];childbirth experience was also negatively affected [15].

In our centre, women who presented in latent phase of labor were typically offered hospitalization. For term women, if active labor had not occurred after an overnight stay and contractions persisted, management the following morning depended on provider and patient preference, with labor induction more likely at full term compared to expectant management.

In a 2014 trial report on 129 nulliparous women with prolonged latent phase of labor, the Cesarean section rate was 15/65 (23.1%) with early induction and 24/64 (37.5%) with expectant management *P* = 0.076 (power 45%) [16]. We undertook a powered study based on the aforementioned findings to evaluate labor induction compared to expectant management on Cesarean delivery rate as primary outcome in nulliparas who are at higher risk of Cesarean delivery with labor induction [17].

## Methods

This was a randomized controlled trial conducted in a University hospital in Malaysia, with the first participant recruited on 5 June, 2015 and the last on 10 November 2017. Our delivery unit was located within a tertiary referral facility with about 5000 deliveries per year and an overall Cesarean delivery rate of 30%. The trial was approved by the Medical Ethics Committee of University Malaya Medical Center (date of approval: 25 February 2015; reference number: 20151–971) and registered in the online searchable Malaysian National Medical Research Register (no. NMRR-15-16-23,886; https://www.nmrr.gov.my) on 6 January 2015 before trial enrolment. Malaysian research regulations governing public health institutions requires NMRR registration after a ‘preliminary’ ethics approval with ethics approval formalized after the issue of an NMRR number. In addition, the trial was also registered in the International Standard Randomised Controlled Trials Number registry, registration number ISRCTN14099170 (http://www.isrctn.com/ISRCTN14099170) on 5 Nov 2015 as Malaysian NMRR might be unrecognized. The trial was conducted in accordance with the Declaration of Helsinki on human experimentation. The study adhered to CONSORT guidelines.

### Participants

Inclusion criteria were prolonged latent phase of labor (defined as an overnight hospitalization of at least 8 h for latent phase of labor), persistent contractions of at least 1 in 30 min, cervical dilation ≤3 cm and intact membranes, nulliparous (no prior pregnancy > 20 weeks), a singleton fetus, cephalic presentation, reassuring fetal heart rate tracing and ≥ 39 weeks’ gestation. Exclusion criteria included known fetal abnormalities, estimated fetal weight ≥ 4 kg or ≤ 2 kg (clinical assessment for small or large for gestational age, if either suspected then ultrasound estimation of fetal weight), contraindications to expectant management (e.g. pregnancy induced hypertension, suspected abruptio), previous uterine surgery (e.g. myomectomy or hysterotomy), known prostaglandin allergy or contraindication to vaginal delivery.

Eligible women were approached, provided with the patient information sheet, verbally counselled, and written informed consent were taken from women who agreed to participate by co-investigators LLMB and subsequently PS on investigator availability. Pre-intervention assessments were Bishop scoring by care provider, fetal heart rate tracing, and visual numerical rating scale (VNRS 0 to 10, higher score more pain) contraction pain score. Participants’ relevant demographic and clinical data were transcribed onto the Case Report Form.

### Randomization and allocated intervention

Participants were randomized to induction of labor or expectant management (for at least 24 h or until indicated intervention according to care provider) by the opening of the lowest numbered sealed, numbered and opaque envelop remaining. Envelopes were prepared using a computer generated random sequence using random.org by an investigator who was not involved in the trial recruitment.

Induction of labor was undertaken according to care provider preference taking into account cervical favorability and frequency of contractions. Common methods we used were amniotomy with or without immediate titrated oxytocin infusion, titrated oxytocin infusion [18] or vaginal dinoprostone pessary [19].

In the expectant management group, spontaneous onset of active phase of labor (defined as regular contractions with cervical dilation > 4 cm) was awaited for at least 24 h; active management including labor induction might be carried based on provider and patient consensus thereafter.

### Sample size

A previous trial has shown Cesarean delivery rates of 23.1% vs 37.5% in the early induction group vs. the expectant management group [16]. Applying alpha 0.05, power 80%, 1 to 1 trial arm ratio and applying the Chi Square test, 159 patients were needed in each arm.

### Outcomes measures

The primary outcome was Cesarean delivery. Secondary maternal outcomes were labor duration outcomes (intervention to active phase of labor i.e. cervical dilation ≥5 cm and to delivery), epidural analgesia needed, intrapartum oxytocin, uterine hyperstimulation syndrome (non-reassuring fetal heart tracing concurrent with uterine tachysystole ≥6 contraction in 10 min), postpartum hemorrhage (blood loss ≥500 ml) and patient’s satisfaction (Likert response) to allocated intervention, during delivery and baby outcome obtained after delivery before hospital discharge. Secondary neonatal outcomes were 5-min Apgar score below 7, cord artery metabolic acidosis (pH ≤ 7 and BE [base excess] ≤ − 8 mmol/L), birth weight and admission to the neonatal intensive care unit (NICU) and admission indications.

### Statistical analysis

Data were entered into a statistical software package SPSS (Version 23, IBM Corp, Armonk, NY). The Student *t* test was used to analyse means and continuous data and chi-square test for categorical data. Two-sided *P* values were reported, and a P value of < 0.05 for all variables was regarded as significant.

### Ethical aspects

Women who chose not to participate received standard care and participants who decided to withdraw may do so without having to give a reason and their care was not affected. Participants were not remunerated.

## Results

**Fig. 1:**
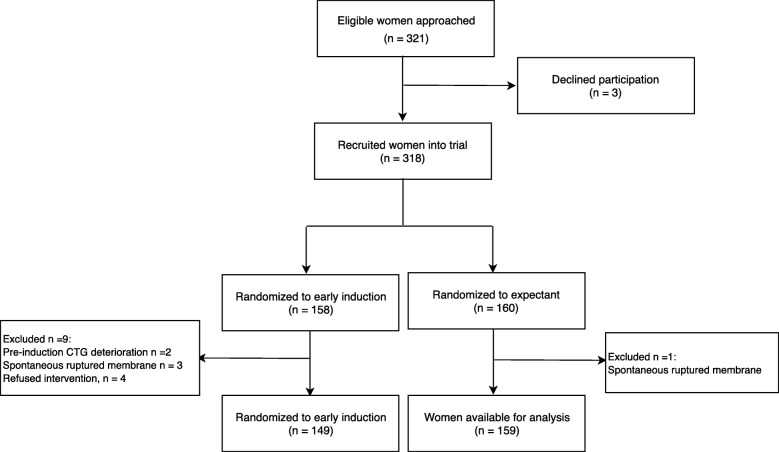
Recruitment flow chart into a randomized trial of induction of labor compared to expectant management in term nulliparas with a latent phase of labor of more than 8 hours

Figure 1 depicts the recruitment flow of trial participants. Of 321 eligible women approached, 3 declined participation; 318 women were randomized (158 to labor induction and 160 to expectant management). Due to criteria infringements, we excluded nine women allocated to labor induction due to 1) labor induction refusal (four women), 2) membranes already ruptured (three women) and 3) pre-induction non-reassuring fetal heart rate tracing (two women) and one woman assigned to expectant management due to membranes already ruptured.

Table 1 depicts the characteristics of the participants stratified according to randomization to early induction or expectant management. Characteristics including of the Bishop score were similar across trial arms.

**Table 1:** Characteristics of trial participants

Characteristics	Induction (*n* = 158)	Expectant (*n* = 160)	*P* value
Age (years)	28.0 ± 3.9	27.6 ± 3.4	0.31
Gestational age (weeks)	39.9 ± 0.5	39.8 ± 0.5	0.18
Body mass index (BMI)	27.3 ± 4.9	27.7 ± 3.5	0.39
Ethnicity			0.40
Malay	100 (63.3)	114 (71.3)	
Chinese	28 (17.7)	21 (13.1)	
Indian	21 (13.3)	12 (7.5)	
Others	9 (5.7)	13 (8.1)	
Hours of contraction pain at recruitment	12 [10–20]	12 [9–19]	0.97
Cervical Bishop score at recruitment	4.3 ± 1.6	4.1 ± 1.4	0.36
Contraction pain score (VNRS)^a^ at recruitment	6.5 ± 1.9	6.6 ± 1.7	0.69

Data expressed as mean ± standard deviation, median [interquartile range] or number (%). Analyses by Student *t* test for continuous variables and Chi Square test for categorical data sets

^a^11-point verbal numerical rating scale (VNRS) with 0 representing no pain and 10 representing worst possible pain

Table 2 depicts the primary outcomes analysis: Cesarean delivery (vs. vaginal delivery) rates were 24.2% (36/149) vs. 23.3% (37/159) RR 1.0 95% CI 0.7–1.6, *P* = 0.96; the spontaneous vaginal delivery rates 55.0% (82/149) vs. 56.6 (90/159), instrumental (forceps or vacuum) vaginal delivery rates were 20.8% (31/149) vs. 20.1% (32/159), *P* = 0.88 (3 by 2 Chi Square analysis) for labor induction vs. expectant arms respectively. Cesarean delivery indications were also similar across trial arms.

**Table 2:** Primary Outcomes

Characteristics	Induction (*n* = 149)	Expectant (*n* = 159)	RR (95% CI)	*P* value
Primary Outcome				
Cesarean delivery	36 (24.2)	37 (23.3)	1.0 (0.7–1.6)	0.96
Vaginal delivery	113 (75.8)	122 (76.7)		
Spontaneous vaginal delivery	82 (55.0)	90 (56.6)		0.88
Instrumental vaginal delivery	31 (20.8)	32 (20.2)		
Indications of Cesarean	*n* = 36	*n* = 37		0.65
Non-reassuring fetal status	16 (44)	19 (51)		
Failure to progress in labor	18 (50)	15 (41)		
Failed induction of labor	2 (6)	3 (8)		

Data expressed as number (%). Analyses by Chi Square test for categorical data, two-sided *P* < 0.05 as significant

Table 3 depicts the secondary maternal and Table 4 the neonatal outcomes. Intervention to delivery as expected were significantly shorter in the induction of labor arm 17.1 ± 9.9 vs 40.1 ± 19.8 h *P* < 0.001, with both the intervention to active labor and active labor to delivery intervals also significantly shorter in the labor induction arm 9.6 ± 10.2 vs 29.6 ± 18.5 h *P* < 0.001 and 7.6 ± 3.6 vs. 10.5 ± 7.2 h *P* < 0.001 respectively. Intervention to hospital discharge interval was also significantly shorter in the labor induction arm 2.4 ± 1.2 vs. 2.9 ± 1.4 days *P* < 0.001 but the delivery to hospital discharge is significantly longer 1.4 ± 1.2 vs. 1.0 ± 1.3 days *P* = 0.03. As expected the use of vaginal dinoprostone for labor induction was higher in the labor induction arm 19.5% (29/149) vs 8.2% (13/159) RR 2.4 95% CI 1.3–4.4 *P* = 0.01 but oxytocin use in labor was only not significantly higher 55.7% (83/149) vs 47.8% (76/159) RR 1.4 95% CI 0.9–2.2 *P* = 0.17. Other maternal outcomes of intrapartum epidural analgesia, uterine hyperstimulation syndrome, postpartum hemorrhage and Likert scale satisfaction responses on allocated intervention, labor and delivery and baby outcomes were similar across trial arms. Secondary neonatal outcomes of Apgar score at 5 min, umbilical cord artery pH and base excess, birth weight, neonatal admission and indications were not significantly different across trial arms.

**Table 3:** Secondary Outcomes

Outcomes	Induction (*n* = 149)	Expectant (*n* = 159)	RR (95%CI)	*P* value
Maternal Outcomes				
Duration of labor				
Intervention to delivery	17.1 ± 9.9	40.1 ± 19.8		< 0.001
Intervention to active phase of labor	9.6 ± 10.2	29.6 ± 18.5		< 0.001
Active labor^a^ to delivery	7.6 ± 3.6	10.5 ± 7.2		< 0.001
Prostaglandin use	29 (19.5)	13 (8.2)	2.4 (1.3–4.4)	0.01
Epidural in labor	20 (13.4)	20 (12.6)	0.9 (0.5–1.8)	0.87
Oxytocin use in labor	83 (55.7)	76 (47.8)	1.4 (0.9–2.2)	0.17
Hyper-stimulation syndrome	0	0		
Post-partum hemorrhage (≥ 500 ml)	8 (5.4)	8 (5.0)	1.1 (0.4–2.9)	1.0
Intervention to discharge interval (days)	2.4 ± 1.2	2.9 ± 1.4		< 0.001
Delivery to discharge interval (days)	1.4 ± 1.2	1.0 ± 1.3		0.03
Likert scale responses				
Satisfaction on allocated intervention			1.1 (0.9–1.2)	0.54
Satisfied^b^	105 (70.5)	106 (66.7)		
Not satisfied^b^	44 (29.5)	53 (33.3)		
Satisfaction on delivery			0.9 (0.7–1.0)	0.07
Satisfied^b^	91 (61.1)	113 (71.1)		
Not satisfied^b^	58 (38.9)	46 (28.9)		
Satisfaction on pregnancy outcomes			0.9 (0.8–1.1)	0.31
Satisfied^b^	103 (69.1)	119 (74.8)		
Not satisfied^b^	46 (30.9)	40 (25.2)		

Data expressed as mean ± standard deviation or number (%). Analyses by Student *t* test for continuous variables and Chi Square test for categorical data sets

^a^Active labor identified when cervical dilatation ≥4 cm

^b^Recategorization of Likert scale responses: “satisfied” includes fully satisfied or somewhat satisfied; “Not satisfied” includes neither satisfied nor dissatisfied, somewhat dissatisfied, and strongly dissatisfied

**Table 4:** Secondary Outcomes

Outcomes	Induction (*n* = 149)	Expectant (*n* = 159)	RR (95%CI)	*P* value
Neonatal outcomes				
Apgar score at 5 min	9.1 ± 0.8	9.1 ± 0.7		0.50
Apgar score ≤ 7 at 5 min	3 (2.0)	7 (4.4)	0.5 (0.1–1.8)	0.34
Cord blood pH	7.3 ± 0.1	7.3 ± 0.1		0.35
pH ≤ 7	2 (1.3)	3 (1.9)	0.7 (0.1–4.3)	1.00
Base excess ≤ −8	2 (1.3)	6 (3.8)	0.4 (0.1–2.0)	0.30
Birth weight	3.1 ± 0.3	3.1 ± 0.4		0.24
Admission to neonatal unit	6 (3.9)	9 (5.6)	0.7 (0.2–2.0)	0.60
Neonatal tachypnoea	1	1		
Neonatal Jaundice^a^	2	3		
Observation	0	3		
Others^b^	3	2		
Neonatal antibiotics	1	1		

Data expressed as mean ± standard deviation or number (%). Analyses by Student *t* test for continuous variables and Chi Square test for categorical data sets

^a^Neonatal jaundice defined as jaundice requiring phototherapy at maternal bedside

^b^Others include: 1 congenital heart disease, 1 hypoglycemia, 1 imperforated anus and 2 pneumonia

### Sensitivity analysis

Post randomization, there were nine exclusions in the labor induction arm, and one in the expectant management arm. Assuming worst case scenario that all nine excluded women in the labor induction arm had Cesarean delivery and the excluded woman in the expectant arm had a vaginal delivery, the Cesarean delivery rates across trial arms were still not significantly different; 45/158 (28.4%) vs. 37/160 (23.1%) *P* = 0.29.

## Discussion

Our trial did not demonstrate a reduction in Cesarean delivery rate in the labor induction arm; the point estimate for Cesarean delivery marginally favored the expectant management arm in contrast to our hypothesis. The point estimate from our data in the opposite direction to our hypothesis indicate that a further increase in sample size is unlikely to bolster support for our hypothesis derived from Brane et al [16] and hence futile. A retrospective study (which did not adjust for risk characteristics) shows that nulliparas having a prolonged latent phase and remaining in hospital had fewer spontaneous vaginal births, more emergency CSs and more babies with Apgar < 7 at 5 min compared with those returning home [20] potentially inconsistent to the finding that women indicating that they had been in labour for 24 h or longer at the time of hospital admission were at elevated risk for cesarean birth and meconium-stained amniotic fluid [21] and a stark contrast to the beneficial interventionist approach of Brane et al. [16]

The recent Arrive trial of labor induction at 39 weeks’ gestation vs. expectant management in low risk nulliparas showed a significant reduction in Cesarean delivery rate, pain at delivery, greater perceived control during childbirth but a non-significant reduction in composite neonatal morbidity with labor induction [22]. Our trial finding of very similar Cesarean delivery rate and similar patient satisfaction with early labor induction is in contrast to the Arrive trial [22] but our and their populations’ presentations were quite different albeit all were nulliparas; our trial is not powered to evaluate neonatal outcomes.

In the seminal TermProm trial, amongst nulliparas, Cesarean delivery rates were 14.1% vs 13.7% (induction with oxytocin vs expectant management then oxytocin induction if required) and 13.7% vs. 15.2% (induction with prostaglandin management vs. expectant management then prostaglandin induction if required) [23]: the Cesarean delivery rates for labor induction and expectant management were very similar, consistent with our finding in nulliparas with prolonged latent phase of labor. However our overall Cesarean delivery rate of 24% is higher than the 14% overall rate in the TermProm trial. Cesarean delivery rates were 23.1% vs 37.5% for labor induction group vs. expectant management for prolonged latent phase of labor participants in Brane et al. [16].

A study from our centre on the labour induction of nulliparas at term with unfavorable cervixes demonstrated that maternal satisfaction is associated with a shorter induction-to-delivery interval [24]. A very recent study also found that women’s perception of quality of intrapartum care, the birth experience and feelings were related to length of the latent phase of labour [25]. In this trial, despite significantly shorter intervention to delivery (both components of intervention to active labor and active labor to delivery intervals being significantly shorter when considered separately), women assigned to labor induction were not significantly more satisfied with their allocated intervention assessed to delivery and on baby outcome; indeed the point maternal satisfaction point estimates to delivery and on baby outcome non-significantly favored the expectant management arm. The lack of a significant correlation of maternal satisfaction to intervention to delivery interval in this trial was unexpected and seemingly difficult to understand as intervention to active labor was prolonged by a considerable mean 23 h with expectant management but satisfaction may be counterbalanced by the significantly longer delivery to hospital discharge in the labor induction arm. It should also be noted that 4/158 (2.5%) of women randomized to labor induction refused their allocated intervention and withdrew from the study which indicated that a small number of women showed resistance to labor induction compared to letting events take their natural course when contractions had already started. However, assessing satisfaction is a complex process and we had not covered these secondary satisfaction outcomes comprehensively with the use of one-dimensional questions. A recent qualitative study shows that nulliparas with a prolonged latent phase of labor preferred woman-centred care with midwives playing an important role in support and the need for support increases as the time spent in latent phase increases [26]. A recent metasynthesis of the literature on first-time mothers’ experiences of early labour suggested that women’s needs when planning a hospital birth were not being adequately met at this stage of the labour process. These considerations which were not covered in our trial could plausibly influence participants’ satisfaction.

In our centre, the overall Cesarean delivery rate after “cold” labor induction of nulliparas with unfavorable cervixes was 42–45% [24]. The 23–27% Cesarean delivery in this trial indicates that our current population of women in prolonged latent phase was in a lower risk group for Cesarean delivery than in “cold case” nulliparous labor induction probably due to a generally more favorable cervix at the start, plausibly to a maternal environment more amenable to respond well to induction of labor and comprising healthier women who did not have a conventional indication for labor induction at their presentation and hospital admission.

### Strengths and limitations

As to strength of our trial, Brane et.al [16] defined prolonged latent phase as presence of contraction (based on women’s perception) for more than 18 h with cervical dilatation of 4 cm or less. Our definition of prolonged latent phase of labor is defined by persistent contraction after an overnight hospital stay, a more practical categorization as in routine practice, care planning and decision making are usually undertaken at the morning round. Our study is powered to Brane et al’s finding [16] but differences in inclusion criteria might have contributed to our trial’s finding of similar Cesarean delivery rates across our trial arms in contrast to theirs that favored labor induction.

As to limitations, we were not able to mask interventions due to intrinsic nature of the interventions. We had a small number of post randomization drop-outs which reduced power; however sensitivity analysis assuming worst case scenario did not materially change the finding of no significant difference in primary outcome Cesarean delivery rate across trial arms. Our trial population might also represent a number of mislabelled “prolonged latent phase of labor” who might actually be cases of false labour or in normal latent phase as our qualifying threshold duration for uterine contractions could be shorter than Friedman’s 20 h [1, 8]. Any labor induction in the expectant management arm soon after the 24 h of prohibition might have reduced power as the difference between the interventions would be less distinct. Participants in the trial were probably a minority of potentially eligible women through our trial enrolment period as our screening and recruitment process was not comprehensive with the possibility for recruitment bias.

## Conclusion

Nulliparas at full term in prolonged latent phase of labor should be informed that labor induction compared with expectant management is associated with a significantly shorter intervention to delivery interval but Cesarean delivery, patient satisfaction and neonatal outcomes are not significantly different compared to expectant management to aid their decision making on management.

## Data Availability

All data generated or analysed during this study are included in this published article and the datasets used are available from the corresponding author on reasonable request.

## Abbreviations

BE1: Base excess
NICU: Neonatal intensive care unit
UK: United Kingdom
